# Intentional use of both opioids and cocaine in the United States

**DOI:** 10.1016/j.pmedr.2023.102227

**Published:** 2023-05-05

**Authors:** Xiguang Liu, Mendel E. Singer

**Affiliations:** Case Western Reserve University, Dept. of Population and Quantitative Health Sciences, 10900 Euclid Ave, SOM WG-57, Cleveland, OH 44106, United States

**Keywords:** Opioid, Fentanyl, Cocaine, Polysubstance, Substance abuse, Overdose

## Abstract

The combination of opioids and cocaine has been increasingly implicated in overdose fatalities, but it is unknown how much is intentional vs. fentanyl-adulterated drug supply. 2017–2019 data from the nationally representative National Survey on Drug Use and Health (NSDUH) was used. Variables included sociodemographics, health, and 30-day drug use. Opioid use captured heroin, and prescription pain reliever use not according to own doctor. Modified Poisson regressions were used to estimate prevalence ratios (PRs) for variables associated with opioid and cocaine use. Among the 167,444 responders, 817(0.49%) reported use of opioids on a regular or daily basis. Of these, 28% used cocaine ≥1 of prior 30 days, 11% >1 day. Of 332(0.20%) who used cocaine on a regular/daily basis, 48% used opioids ≥1 of prior 30 days, 25% >1 day. People with serious psychological distress were >6 times as likely to use both opioids and cocaine regularly/daily (PR = 6.48; 95% CI = [2.82–14.90]) and people who have never been married were 4 times as likely (PR = 4.17; 95% CI = [1.18–14.75]). Compared to those living in a small metropolitan region, people living in a large metropolitan region were >3 times as likely (PR = 3.29; 95% CI = [1.43–7.58]) and the unemployed were twice as likely (PR = 1.96; 95% CI = [1.03–3.73]). People with post-high school education were 53% less likely to use opioids and cocaine at least occasionally (PR = 0.47; 95% CI = [0.26–0.86]). People who use opioids or cocaine commonly choose to use the other. Knowing the characteristics of those most likely to use both should guide interventions for prevention and harm reduction.

## Highlights

• Half of regular/daily cocaine users knowingly used opioids in the prior 30 days.
• 1 in 4 regular/daily opioid users knowingly used cocaine in the prior 30 days.
• Use of opioid and cocaine was 6x as common in those with psychological distress.
• Use of opioid and cocaine more common: never married, unemployed, large metro area.
• Use of opioid and cocaine was half as common in people with post-HS education.

## 1 Introduction

### 1.1 History of opioid use

The United States opioid crisis started in the late 1990s when healthcare providers prescribed opioid pain relievers under the false impression they were not addictive (National Institute on Drug Abuse, 2020). The early 2010′s saw a growing number of heroin deaths; followed by a huge spike in overdose deaths due to fentanyl (Centers for Disease Control and Prevention, 2018). Low price; strong potency, and an easy synthetic process are the main reasons for the high prevalence of fentanyl (Kilmer et al., 2014). Yet; overdose deaths in Europe remain low and dominated by heroin, despite increasing use of synthetic opioids (European Monitoring Centre for Drugs and Drug Addiction, 2019). India and China are among the countries with the highest prevalence of heroin use; without a move to illicitly manufactured fentanyl (Taylor et al., 2021). While Australia saw a large increase in fentanyl-related deaths from 2001 to 2014; this rate declined from 2015 to 2021 (Roxburgh and Nielsen, 2022 Nov). Canada; however, has seen a persistent rise. By 2015–2016, opioid-related deaths had moved from prescription opioids to illicit synthetic opioids (Fischer et al., 2019). Since 2020 more than 75% of opioid deaths have involved fentanyl (Government of Canada Special Advisory Committee on the epidemic of opioid overdoses, 2022).

### 1.2 Move to polysubstance use

More recent years have seen a move in the US toward polysubstance use and changes in demographics. Between 2014 and 2015, toxicology reports in Massachusetts indicated that 36% of opioid-related deaths involved opioids with stimulants (cocaine and amphetamines), and 46% mixed opioids with non-stimulant substances (Barocas et al., 2019). A study in Tennessee confirmed the increasing trend of polysubstance use (Nechuta et al., 2018). From 2013 to 2016, prevalence of cocaine among fentanyl overdoses doubled from 9.4% to 18.8% In 25 states, at least one non-opioid drug was involved in 62.6% of all opioid deaths during the January–June period in 2018 (Gladden et al., 2019). By 2018 it became the most prevalent co-occurring cause of death drug in opioid overdoses (Gladden et al., 2019). A study in Ohio found that opioid overdose deaths in 2017 typically involved three or more cause of death drugs, and 37% also had cocaine as a cause of death drug (Bhullar et al., 2022). There is also evidence that a majority of prescription opioid misuse is actually polysubstance misuse (Grigsby and Howard, 2019).

### 1.3 Opioids and cocaine

The increase in overdose deaths involving both opioids and cocaine has changed the demographics of the opioid epidemic. Opioid overdose had been most common in non-Hispanic white males aged 25–55 years old (Gladden et al., 2016). Among Massachusetts unintentional overdose decedents, non-Hispanic blacks are twice as likely to die with opioids and stimulants (cocaine or amphetamines) than with opioids alone compared with non-Hispanic whites (Barocas et al., 2019). Though opioids are the most common type of drug overdose death, cocaine-related overdose death rates in non-Hispanic blacks were about the same as opioid-related overdose death rates in non-Hispanic whites (Shiels et al., 2018). Young age, post high school education, non-rural residency, and mental disorders are other risk factors of opioids overdose (Nechuta et al., 2018). as well as polysubstance opioid overdose (Barocas et al., 2019). Among people who use illicit drugs, stress is the strongest predictor in the study among all possible drug use leading factors (Preston and Epstein, 2011).

A common question is whether decedents intentionally used both opioids and cocaine, or if they were unaware they had a mixture. It is essential to know more about people who intentionally use both opioids and cocaine. A prior study reports on the use of both heroin and cocaine from 2002 to 2017 using the National Survey on Drug Use and Health (Goodwin et al., 2021). This study uses the same survey, but for more recent years, to explore the frequency of cocaine use in people who use opioids and vice versa, and identify risk factors for using both. This update to include all opioids and not just heroin is critical since the opioid overdose fatalities is no longer driven by heroin.

## 2 Methods

### 2.1 Data source

Data from the National Survey on Drug Use and Health (NSDUH) for 2017–2019 was downloaded from the Substance Abuse and Mental Health Services Administration (SAMHSA) website. The NSDUH uses a 5-stage complex survey design. NSDUH targets the civilian, noninstitutionalized (including group quarters) population age ≥12, but omits people with no fixed address (Substance Abuse and Mental Health Services Administration., 2019). In-person interviews employ procedures to assure confidentiality and increase respondents’ willingness to respond honestly to sensitive topics, e.g. use of Computer-assisted interviewing (CAI). The most recent year of data, 2020, lacked the variables on opioid use to be part of this study. NSDUH is a nationally representative survey whose purpose is to estimate the U.S. prevalence of substance use and mental health issues over time, while assessing the consequences and identifying risk factors (Substance Abuse and Mental Health Services Administration., 2019). All respondents age 18 or over were eligible for this study. As this data set was anonymous, publicly available data, this study was ruled to be not human subjects research and therefore exempt from Institutional Review Board (IRB) review.

### 2.2 Variables

Variables in the sociodemographic category included: age, imputation revised gender (biological sex), minority, marital status, education, low income (less than 2 times the Federal Poverty Level), metropolitan status (large/medium/non-metropolitan), and employment status. Overall health was captured as self-assessed overall health and dichotomized to Fair/Poor vs. Excellent/Very Good/Good. Variables in the mental health/distress category included: major depressive episode in prior year, received outpatient mental health treatment in past year, received inpatient mental health treatment in past year, received prescribed medication for mental health treatment in past year, serious psychological distress in past year. All recoded and imputation revised variables are original NSDUH variables.

Drug use was broken down into two components: which drug (opioids, cocaine, both) and frequency of use in the last 30 days. The opioid variable represents either use of heroin or use of a prescription pain reliever in ways other than instructed by their own doctor. Number of days of drug use in the prior 30 days was broken down into the following groups: No use (0), one-time use (1), occasional use (2–4), regular use (5–19), daily use (20–30).

### 2.3 Statistical analysis

Data cleaning and analysis were performed using R version 3.6.3 and Survey package version 4.0 (R: A language and environment for statistical computing. R Foundation for Statistical, Computing [computer program], 2020, Lumley, 2022). Analyses accounted for the complex sampling design.

Bivariable analyses were conducted to find respondent characteristics associated with frequency of drug use. Potential associations were tested using a chi-square test.

Regression analyses were conducted using all responder characteristics as study variables. The primary outcome variables were use of both opioids and cocaine defined in two ways: regular or daily use of both opioids and cocaine, and occasional/regular/daily use of both drugs. Regressions were also run for regular or daily use of one drug in the subset of respondents that used the other drug regularly or daily. Then the regression was rerun expanding use to occasional, regular or daily. For each model, both a logistic regression and a modified Poisson regression was run. The former generated odds ratios, while the latter produced the more intuitive prevalence ratios (PR). PR’s represent how much more or less often a characteristic was found in people with the outcome variable and is the cross-sectional data equivalent of a relative risk. In addition to producing the more easily interpreted prevalence ratios as opposed to odds ratios, modified Poisson regression is also more reliable and preferable to logistic regression because it is less likely to make an overestimate (Chen et al., 2018, Zou, 2004). Therefore, while the odds ratios from the logistic regressions are included in the tables, the narrative will only discuss the prevalence ratios.

To avoid overfitting the model, we used the rule of 10 observations per person in the smaller outcome category. When the model would otherwise be overfit, best subset selection was implemented for the logistic regression model and the study variables in that best subset were used in the corresponding modified Poisson regression. The R function Bestglm (bestglm, n.d.) was used with the exhaustive method applied to select subsets for binomial family distributions. The best subset was chosen based on the smallest value of the Bayesian information criterion (BIC).

## 3 Results

### 3.1 Descriptive statistics

There were a total of 167,444 adults who responded to the questions on drug use. All data is self-reported. There were 817 (0.49%) who used opioids on a regular or daily basis. Of these, 225 (28%) used cocaine on at least one day in the preceding 30 days and 86 (11%) used cocaine on two or more days. There were 332 (0.20%) who regularly or daily used cocaine. Of these, 161 (48%) used opioids at least one day in the prior 30 days and 84 (25%) used opioids at least occasionally (at least two of the past 30 days).

Patient characteristics were broken down by use of opioids (Table 1) and cocaine (Table 2). Many similar effects were found for each substance. Male sex, age18-34, lack of post-high school education, living in poverty, not married, poor overall health were associated with increased drug use. All mental health related variables were associated with increased use of opioids and increased use of cocaine: Whites were associated with heavier use of opioids. For cocaine, whites were more likely to use cocaine infrequently (2–4 times in past 30 days) and less likely to use cocaineregularly or daily. Residents in large metropolitan areas used less opioids but more cocaine. The unemployed were more likely to use opioids at each level of use, while being less likely to use cocaine infrequently (2–4 times in past 30 days) but more likely to use regularly or daily.

**Table 1:** Opioid Use in Prior 30 Days by Sociodemographics and Health: U.S. Adults, 2017–2019.

	No Use N = 160,589 (95.71%)	One-Time Use N = 5,698 (3.40%)	Occasional Use N = 661 (0.39%)	Regular Use N = 521 (0.31%)	Daily Use N = 322 (0.19%)	p-value
Sex						< 0.01
Female	83,918 (52.3%)	2,827 (49.6%)	360 (54.5%)	233 (44.7%)	151 (46.9%)	
Male	76,671 (47.7%)	2,871 (50.4%)	301 (45.5%)	288 (55.3%)	171 (53.1%)	

Age						< 0.01
18–34	63,608 (52.2%)	3,207 (66.6%)	352 (61.3%)	291 (62.6%)	190 (61.3%)	
35–49	32,481 (26.7%)	1,140 (23.7%)	154 (26.8%)	113 (24.3%)	87 (28.1%)	
50–64	14,325 (11.8%)	329 (6.8%)	54 (9.4%)	52 (11.2%)	29 (9.4%)	
65 and over	11,390 (9.4%)	136 (2.8%)	14 (2.4%)	9 (1.9%)	4 (1.3%)	

Race/Ethnicity						< 0.01
Minority	67,508 (42.0%)	2,257 (39.6%)	272 (41.1%)	184 (35.3%)	81 (25.2%)	
Non-Hispanic White	93,081 (58.0%)	3,441 (60.4%)	389 (58.9%)	337 (64.7%)	241 (74.8%)	

County Status						<0.01
Large Metro.	72,343 (45.0%)	2,479 (43.5%)	280 (42.4%)	215 (41.3%)	131 (40.7%)	
Small Metro.	56,331 (35.1%)	2,119 (37.2%)	242 (36.6%)	199 (38.2%)	117 (36.3%)	
Non-Metro.	31,915 (19.9%)	1,100 (19.3%)	139 (21.0%)	107 (20.5%)	74 (23.0%)	

Education						< 0.01
Post High School	74,705 (61.3%)	2,751 (57.2%)	319 (55.6%)	221 (47.5%)	128 (41.3%)	
H.S Grad. Or Less	47,099 (38.7%)	2,061 (42.8%)	255 (44.4%)	244 (52.5%)	182 (58.7%)	

Low Income						< 0.01
Yes	46,381 (38.5%)	2,163 (45.5%)	285 (50.2%)	238 (51.3%)	170 (54.8%)	
No	74,176 (61.5%)	2,593 (54.5%)	283 (49.8%)	226 (48.7%)	140 (45.2%)	

Marital Status						< 0.01
Married	50,868 (35.9%)	1,278 (23.5%)	171 (26.7%)	98 (19.6%)	72 (22.6%)	
Previously Married	16,464 (11.6%)	596 (11.0%)	89 (13.9%)	75 (15.0%)	49 (15.4%)	
Never Been Married	74,320 (52.5%)	3,555 (65.5%)	380 (59.4%)	328 (65.5%)	197 (61.9%)	

Employment Status						< 0.01
Employed	83,072 (68.2%)	3,215 (66.8%)	378 (65.9%)	268 (57.6%)	156 (50.3%)	
Unemployed	38,732 (31.8%)	1,597 (33.2%)	196 (34.1%)	197 (42.4%)	154 (49.7%)	

Overall Health						< 0.01
Excellent/V. Good/Good	145,311 (90.5%)	4,862 (85.3%)	529 (80.0%)	387 (74.3%)	231 (71.7%)	
Fair/Poor	15,237 (9.5%)	835 (14.7%)	132 (20.0%)	134 (25.7%)	91 (28.3%)	

Mental Health in Past Year						
Outpatient Treatment	10,348 (8.5%)	788 (16.6%)	107 (18.9%)	94 (20.5%)	56 (18.2%)	< 0.01
Major Depressive Episode	10,918 (9.0%)	1,044 (21.7%)	148 (25.8%)	123 (26.5%)	87 (28.1%)	< 0.01
Prescription Medication	15,341 (12.6%)	1,110 (23.3%)	169 (29.8%)	136 (29.5%)	80 (25.9%)	< 0.01
Psychological Distress	18,671 (15.3%)	1,741 (36.2%)	229 (39.9%)	216 (46.5%)	144 (46.5%)	< 0.01
Inpatient Treatment	1,269 (1.0%)	184 (3.9%)	23 (4.1%)	38 (8.2%)	12 (3.9%)	< 0.01

**Table 2:** Cocaine Use in Prior 30 Days by Sociodemographics and Health: U.S. Adults, 2017–2019.

	No Use N = 164,372 (97.64%)	One-Time Use N = 3,164 (1.88%)	Occasional Use N = 478 (0.28%)	Regular Use N = 250 (0.15%)	Daily Use N = 82 (0.05%)	p value
Sex						< 0.01
Female	86,220 (52.5%)	1,277 (40.4%)	174 (36.4%)	83 (33.2%)	39 (47.6%)	
Male	78,152 (47.5%)	1,887 (59.6%)	304 (63.6%)	167 (66.8%)	43 (52.4%)	

Age						< 0.01
18–34	64,580 (52.0%)	2,475 (82.4%)	380 (81.4%)	175 (71.4%)	43 (52.4%)	
35–49	33,421 (26.9%)	415 (13.8%)	67 (14.3%)	48 (19.6%)	26 (31.7%)	
50–64	14,631 (11.8%)	100 (3.3%)	19 (4.1%)	22 (9.0%)	13 (15.9%)	
65 and over	11,562 (9.3%)	14 (0.5%)	1 (0.2%)	0 (0.0%)	0 (0.0%)	

Race/Ethnicity						< 0.01
Minority	69,202 (42.1%)	1,070 (33.8%)	169 (35.4%)	111 (44.4%)	36 (43.9%)	
NonHisp White	95,170 (57.9%)	2,094 (66.2%)	309 (64.6%)	139 (55.6%)	46 (56.1%)	

County Status						< 0.01
Large Metro.	73,700 (44.8%)	1,563 (49.4%)	250 (52.3%)	135 (54.0%)	51 (62.2%)	
Small Metro.	57,831 (35.2%)	1,108 (35.0%)	168 (35.1%)	76 (30.4%)	21 (25.6%)	
Non-Metro.	32,841 (20.0%)	493 (15.6%)	60 (12.6%)	39 (15.6%)	10 (12.2%)	

Education						< 0.01
Post High school	75,690 (60.9%)	1,974 (65.7%)	311 (66.6%)	122 (49.8%)	35 (42.7%)	
Up to High school	48,504 (39.1%)	1,030 (34.3%)	156 (33.4%)	123 (50.2%)	47 (57.3%)	

Poverty						< 0.01
Live in poverty	47,669 (38.8%)	1,233 (41.5%)	197 (42.5%)	122 (50.0%)	45 (54.9%)	
Not live in poverty	75,253 (61.2%)	1,736 (58.5%)	266 (57.5%)	122 (50.0%)	37 (45.1%)	

Marital Status						< 0.01
Married	52,050 (36.0%)	384 (12.2%)	46 (9.6%)	27 (10.8%)	11 (13.4%)	
Previously Married	16,943 (11.7%)	246 (7.8%)	43 (9.0%)	28 (11.2%)	23 (28.0%)	
Never Been Married	75,758 (52.3%)	2,513 (80.0%)	389 (81.4%)	195 (78.0%)	48 (58.5%)	

Employment Status						< 0.01
Employed	84,303 (67.9%)	2,228 (74.2%)	349 (74.7%)	151 (61.6%)	41 (50.0%)	
Unemployed	39,891 (32.1%)	776 (25.8%)	118 (25.3%)	94 (38.4%)	41 (50.0%)	

Overall Health						< 0.01
Good	148,369 (90.3%)	2,786 (88.1%)	427 (89.3%)	202 (80.8%)	57 (69.5%)	
Poor	15,961 (9.7%)	378 (11.9%)	51 (10.7%)	48 (19.2%)	25 (30.5%)	

Mental Health in Past Year						
Outpatient Treatment	10,826 (8.8%)	441 (14.8%)	66 (14.5%)	40 (16.5%)	17 (21.0%)	< 0.01
Major Depressive Episode	11,526 (9.3%)	644 (21.4%)	83 (17.8%)	50 (20.4%)	17 (20.7%)	< 0.01
Prescription Medication	16,025 (13.0%)	646 (21.7%)	90 (19.6%)	56 (23.1%)	24 (29.6%)	< 0.01
Psychological Distress	19,664 (15.8%)	1,042 (34.7%)	157 (33.6%)	99 (40.4%)	39 (47.6%)	< 0.01
Inpatient Treatment	1,374 (1.1%)	118 (4.0%)	10 (2.2%)	18 (7.4%)	10 (12.3%)	< 0.01

### 3.2 Risk factors for use of both opioids and cocaine

Use of both opioids and cocaine on a regular or daily basis was more common in four groups, as seen by their prevalence ratios (PR) resulting from the modified Poisson regressions (Table 3). People with serious psychological distress in the past year were more than 6 times as likely to be using both opioids and cocaine (PR = 6.48; 95% CI = [2.82–14.90]) and people who have never been married were about 4 times as likely (PR = 4.17; 05% CI = [1.18–14.75]). Compared to those living in a small metropolitan region, people living in a large metropolitan region were over 3 times as likely (PR = 3.29; 95% CI = [1.43–7.58]). The unemployed were nearly twice as likely (PR = 1.96; 95% CI = [1.03 – 3.73]). People with more than a high school education were 58% less likely to be using both opioids and cocaine, but this was borderline statistically significant (p =.0544) (PR = 0.42; 95% CI = [0.18–1.02]).

**Table 3:** Regression analysis of use of opioid and cocaine on a regular or daily basis: U.S. Adults, 2017–2019 (N = 128,319).

	Logistic Regression		Modified Poisson Regression	
	OR	CI	PR	CI
Large Metro.	3.30 **	1.43–7.60	3.29**	1.43–7.58
Non-Metro.	1.44	0.51–4.01	1.44	0.51–4.01
Post High school	0.42	0.18–1.02	0.42	0.18–1.02
Previously Married	1.40	0.33–5.87	1.40	0.33–5.87
Never Been Married	4.18 *	1.18–14.79	4.17 *	1.18–14.75
Unemployed	1.97 *	1.03–3.75	1.96 *	1.03–3.73
Minority	0.41	0.15–1.14	0.41	0.15–1.14
Psychological Distress: Yes	6.50 **	2.83–14.96	6.48**	2.82–14.90

* p < 0.05.

** p <.01.

Results were somewhat different when expanding the outcome variable to include use of both opioids and cocaine even on an occasional basis (Table 4). People who were never married were more than 5 times as likely to use both opioids and cocaine (PR = 5.45; 95% CI = [2.62–11.34]), and those who had been previously married had a borderline statistically significant result (p =.0527): 2.52 times as likely to use both opioids and cocaine (PR = 2.52; 95% CI = 0.99–6.45]). People with a history of serious psychological distress in the past year were 3.69 times as likely (PR = 3.69; 95% CI = [2.14–6.36]). Education beyond high school had a protective effect, being 53% less likely to be using both kinds of drugs (PR = 0.47; 95‘% CI = 0.26–0.86). Living in a large metropolitan area failed to reach statistical significance (p =.0776) despite a 63% increased frequency of use of both opioids and cocaine (PR = 1.63; 95% CI = [0.95 – 2.80]).

**Table 4:** Regression analysis of use of opioid and cocaine on an occasional or regular or daily basis: U.S. Adults, 2017–2019 (N = 127,359).

	Logistic Regression		Modified Poisson Regression	
	OR	CI	PR	CI
Large Metro.	1.63	0.95–2.80	1.62	0.95–2.79
Non-Metro.	1.05	0.56–1.98	1.05	0.56–1.98
Post High school	0.47 *	0.26–0.86	0.47 *	0.26–0.86
Previously Married	2.52	0.99–6.45	2.52	0.99–6.44
Never Been Married	5.45 **	2.62–11.34	5.44 **	2.62–11.31
Unemployed	1.18	0.71–1.95	1.18	0.71–1.95
Minority	0.73	0.40–1.34	0.74	0.41–1.34
Psychological Distress: Yes	3.69 **	2.14–6.36	3.68 **	2.13–6.34
Outpatient Mental Health Treatment: Yes	1.17	0.50–2.76	1.17	0.50–2.75
Prescription Medication: Yes	1.81	0.69–4.72	1.81	0.69–4.70

* p < 0.05.

** p <.01.

### 3.3 People who use opioids or cocaine: Risk factors for use of the other drug

We further explored dual use of opioids and cocaine by examining groups of users of opioids and checking for risk factors for also using cocaine at least occasionally, and vice versa. Among opioid users, the only predictor of cocaine use was living in a large metropolitan area, which was 3.45 times as likely (PR = 3.45; 95% CI = [1.55–7.68]) in the regular or daily opioid users, and 2.27 times as likely (PR = 2.27; 95% CI = [1.26–4.11]) in people using opioids occasionally/regularly/daily. Among cocaine users, serious psychological distress in the past year was associated with 2.10 times the frequency of using opioids (PR = 2.10; 95% CI = [1.09–4.03]) in regular or daily users of cocaine, but was only approaching statistical significance (p =.0862) with 58% more using of opioids in those using cocaine occasionally/regularly/daily (PR = 1.58; 95% CI + [0.93–2.66]). Residents of large metropolitan areas and minorities were nearly statistically significantly associated (p =.0635 and p =.0615, respectively) with opioid use in people who use cocaine on a regular/daily basis. There were nearly double the number using opioids in the large metropolitan areas (PR = 1.93; 95% CI = [0.96–3.89]), and 55% fewer using opioids in minorities (PR = 0.45; 95% CI = [0.20–1.04]). However, these effects were not found when considering people who use cocaine occasionally, and not just regularly/daily.

## 4 Discussion

This is the first study to extensively examine risk factors for use of both opioids and cocaine in a live population, as opposed to using decedent data. During 2010–2019 in New York state, nearly 2/3 of the 1,320 cocaine overdose deaths involved synthetic opioids (Opioid Surveillance Team, 2021.). A CDC study of 24 states and the District of Columbia for the first half of 2019 found that nearly 1/3 of the 16; 236 overdose deaths involved opioids with a stimulant (O'Donnell et al., 2020). A recent study found that people who use cocaine without history of opioid use were not only less aware of the presence of fentanyl in the drug market; but were less likely to carry naloxone or been trained in use of naloxone (Hughto et al., 2022). However; while decedent studies provide important information, they beg the question of whether use of both opioids and cocaine was intentional or not. This study shows that among people who use opioids or cocaine, intentional use of the other is common. Among people regularly using opioids, more than one-fourth had used cocaine in the last month. Among people regularly using cocaine, about half had used opioids at least one day in the prior month and one-fourth had used opioids at least 2 days in the last month. While the overall number of people who use may appear small, capture-recapture studies show the actual prevalence of opioid use disorder to be 4.5 times the estimate from surveys (Barocas et al., 2018). The number of adolescents and adults with opioid use disorder in the US in 2019 was recently estimated at 6.7 – 7.6 million (Keyes et al., 2022). It is likely that both opioid and cocaine use is underestimated here.

This study found a number of risk factors for use of both opioids and cocaine, the greatest being serious psychological distress in the prior year. According to a study on multiple substances, psychological stress was one of the strongest predictors of polysubstance misuse (Preston and Epstein, 2011). Other studies found psychological stress or mental health comorbidity to be a risk factor for opioid misuse (Reyes-Gibby et al., 2016, Han et al., 2017, Chan and Trant, 2018). People with multiple motivations for misuse tended to have higher rates of mental disorders and substance use misbehavior (Schepis et al., 2020a, Schepis et al., 2020b). In a *meta*-analysis qualitative study, Cicero and Ellis summarized several motivations to continue using prescription opioids, and the two most common reasons were related to psychological problems (Cicero and Ellis, 2017).

The second highest risk group for use of both opioids and cocaine was people who have never been married, though there were also signs that people who were previously married also carry increased risk. This has previously been shown to be true in opioid use (Haider et al., 2020).

Living in a large metropolitan region was associated with three times the risk of using both opioids and cocaine. When the misuse frequency is relatively high, on a regular or daily basis, living in a large metro area significantly increases the risk of polysubstance use. Living in a large metropolitan area has previously been shown to be associated with an increasing rate of overdose deaths involving cocaine, among which three-fourths also used an opioid (Nechuta et al., 2018), though another study showed no effect of region on use of nonmedical prescription opioids (Chan et al., 2019).

Higher education was associated with substantially *less* risk of using both opioids and cocaine. This has also been found to be true for decedents who had used both opioids and benzodiazepines (Nechuta et al., 2018). Finally, the unemployed were much more likely to use both opioids and cocaine.

While this survey tells us *who* is using opioids and cocaine, it doesn’t tell us *why*, e.g. to ameliorate the side-effects of the other drug. Our data also does not tell us if both drugs were used simultaneously.

### 4.1 Strengths

This study has several strengths. First, it employed a large, nationally representative dataset. Furthermore, this study consists of a three-year cohort, which increased the sample size sufficiently to draw conclusions about the small proportion of the population that are using both opioids and cocaine. Perhaps the biggest strength is that the data set reflects the living and their chosen drug use, as opposed to prior studies of decedent data, which is available but unlikely to reflect the general population of people who use.

### 4.2 Limitations

First, it is likely there is underreporting of illegal drug use (e.g. social desirability bias) and underrepresentation of people who use drugs (who may not be identified or may be less likely to respond). It is not clear if and how this might impact the relationships between individual characteristics and use of both opioids and cocaine that were found in regression analysis. Furthermore, active military members or institutionalized groups were not included. Though these individuals only composed 3% of the total population in the U.S., they were potentially high-risk for misusing opioids and cocaine (Substance Abuse and Mental Health Services Administration., 2019). Other small, potentially high-risk groups such as the homeless would be hard to reach.

The NSDUH uses opioid misuse, defined as use of heroin or misuse of prescription pain relievers. It is unknown how many respondents would include, for example, illicit fentanyl or analogs, when answering questions about misuse of prescription pain relievers (which includes fentanyl).

Another challenge came from the cross-sectional design of the data, precluding the finding of temporal relationships, such as whether people who currently use both opioids and cocaine tended to have started with opioids and later started taking cocaine as well or vice versa.

Another limitation is that the survey covered frequency of use of different drugs, including opioids and cocaine. However, there was no question identifying if the use of opioids and cocaine was simultaneous.

This study has the opposite limitation of decedent-based studies: data on people who currently use cannot be generalized to overdose decedents. We could not conclude, for example, whether overdose fatalities are more frequently in people who intentionally use both, or if the fatalities are more from cocaine users unaware fentanyl was mixed in. Studies are needed to distinguish overdose risk between those who use both opioids and cocaine knowingly vs. unknowingly.

Finally, this data is from before COVID-19. The pandemic may have impacted polysubstance use, though this study provides the baseline against which it can be compared.

### 4.3 Implications

Fentanyl testing strips may be of limited value if intentional use of both opioids and cocaine is common, though opioid-naïve people intending to use just cocaine would still benefit. Harm reduction, educational efforts and addiction treatment programs aimed at opioid or cocaine use need to consider the other drug as well. Education about, and distribution of naloxone is needed for known cocaine users. The results here suggest that opioid prescribers need to screen patients for cocaine use. Risk factors for use of both opioids and cocaine can be used to guide screening decisions and development of risk scores. Further, through cooperation with other organizations and connecting with additional data records, such as Medicaid, policymakers can target and educate people at risk and prevent the transition from misuse of prescription medicine to illicit substances. However, we may be far from reaching these goals. A recent study of nonfatal overdoses showed that more than 70% of those who had high-dose opioid prescriptions less than 90 days prior to an overdose, had another high-dose opioid prescription with the same prescriber within 90 days after the overdose (Krishnaswami et al., 2022). It is not clear to what extent this suggests the need for increased provider education or reflects poor communication of overdose events to their prescribers. Data connectedness is often a limiting factor. For example, data from Prescription Drug Monitoring Programs (PDMP) can be used for research in some states, but in others information can only be used for the care of that individual and not pooled for generalizable knowledge. Fatality data from medical examiners can usually be freely shared and they are key players in the opioid crisis, but connecting data from emergency medical services (EMS), state nonfatal overdose databases, and hospital electronic health records is limited by issues of anonymity, HIPAA and state law, aside from the lack of standardization of data.

## 5 Conclusions

Intentional use of both opioids and cocaine is common and dual use if not merely the product of adulteration of the drug supply. Risk factors include: psychological stress, not married, living in a large metropolitan area, less education and being unemployed. This information can be used to inform opioid prescribing procedures and guide drug screening and risk score development, as well as drug education, harm reduction and drug treatment programs to address this increasing threat.

## CRediT authorship contribution statement

**Xiguang Liu:** Software, Validation, Formal analysis, Data curation, Writing – original draft, Writing – review & editing, Visualization. **Mendel E. Singer:** Conceptualization, Methodology, Writing – review & editing, Visualization, Supervision, Project administration.

## Declaration of Competing Interest

The authors declare that they have no known competing financial interests or personal relationships that could have appeared to influence the work reported in this paper.

## Data Availability

Data is availabe for free public download. Link provided in manuscript.

## References

[b0005] Barocas J.A., White L.F., Wang J., Walley A.Y., LaRochelle M.R., Bernson D., Land T., Morgan J.R., Samet J.H., Linas B.P. (2018). Estimated Prevalence of Opioid Use Disorder in Massachusetts, 2011–2015: A Capture-Recapture Analysis. Am J Public Health..

[b0010] Barocas J.A., Wang J., Marshall B.D.L., LaRochelle M.R., Bettano A., Bernson D., Beckwith C.G., Linas B.P., Walley A.Y. (2019). Sociodemographic factors and social determinants associated with toxicology confirmed polysubstance opioid-related deaths. Drug Alcohol Depend..

[b0015] *bestglm: Best Subset GLM and Regression Utilities* [computer program]. Version 0.37.32020.

[b0020] Bhullar M.K., Gilson T.P., Singer M.E. (2022). Trends in opioid overdose fatalities in Cuyahoga County, Ohio: Multi-drug mixtures, the African-American community and carfentanil. Drug Alcohol Depend Rep..

[b0025] Centers for Disease Control and Prevention. Understanding the epidemic: Three waves of opioid overdose deaths. 2018; https://www.cdc.gov/drugoverdose/epidemic/index.html. Accessed September 1, 2022.

[b0030] Chan K.T., Trant J. (2018). The Relationship of Psychological Distress and Living with Children and Adolescents for Adult Non-medical Prescription Opioid Users. Child Adolesc Social Work J..

[b0035] Chan K.T., Winston P., Jennings R., Trant J., Moller M. (2019). Age differences in the association of nonmedical prescription opioid use and suicidality. J Opioid Manag..

[b0040] Chen W., Qian L., Shi J., Franklin M. (2018). Comparing performance between log-binomial and robust Poisson regression models for estimating risk ratios under model misspecification. BMC Med Res Methodol..

[b0045] Cicero T.J., Ellis M.S. (2017). The prescription opioid epidemic: a review of qualitative studies on the progression from initial use to abuse. Dialogues Clin Neurosci..

[b0050] European Monitoring Centre for Drugs and Drug Addiction (2019).

[b0055] Fischer B., Pang M., Tyndall M. (2019). The opioid death crisis in Canada: crucial lessons for public health. Lancet Public Health..

[b0060] Gladden R.M., Martinez P., Seth P. (2016). Fentanyl Law Enforcement Submissions and Increases in Synthetic Opioid-Involved Overdose Deaths - 27 States, 2013–2014. MMWR Morb Mortal Wkly Rep..

[b0065] Gladden R.M., O'Donnell J., Mattson C.L., Seth P. (2019). Changes in Opioid-Involved Overdose Deaths by Opioid Type and Presence of Benzodiazepines, Cocaine, and Methamphetamine - 25 States, July-December 2017 to January-June 2018. MMWR Morb Mortal Wkly Rep..

[b0070] Goodwin R.D., Moeller S.J., Zhu J., Yarden J., Ganzhorn S., Williams J.M. (2021). The potential role of cocaine and heroin co-use in the opioid epidemic in the United States. Addict Behav..

[b0075] Government of Canada Special Advisory Committee on the epidemic of opioid overdoses. Opioid- and stimulant-related harms in Canada. Public Health Agency of Canada, Ottawa2022. https://health-infobase.canada.ca/substance-related-harms/opioids-stimulants. Date accessed: October 27, 2022.

[b0080] Grigsby T.J., Howard J.T. (2019). Prescription opioid misuse and comorbid substance use: Past 30-day prevalence, correlates and co-occurring behavioral indicators in the 2016 National Survey on Drug Use and Health. Am J Addict..

[b0085] Haider M.R., Brown M.J., Gupta R.D., Karim S., Olatosi B., Li X. (2020). Psycho-Social Correlates of Opioid Use Disorder among the US Adult Population: Evidence from the National Survey on Drug Use and Health, 2015–2018. Subst Use Misuse..

[b0090] Han B., Compton W.M., Blanco C., Crane E., Lee J., Jones C.M. (2017). Prescription Opioid Use, Misuse, and Use Disorders in U.S. Adults: 2015 National Survey on Drug Use and Health. Ann Intern Med.

[b0095] Hughto J.M.W., Gordon L.K., Stopka T.J., Case P., Palacios W.R., Tapper A., Green T.C. (2022). Understanding opioid overdose risk and response preparedness among people who use cocaine and other drugs: Mixed-methods findings from a large, multi-city study. Subst Abus..

[b0100] Keyes K.M., Rutherford C., Hamilton A., Barocas J.A., Gelberg K.H., Mueller P.P., Feaster D.J., El-Bassel N., Cerdá M. (2022). What is the prevalence of and trend in opioid use disorder in the United States from 2010 to 2019? Using multiplier approaches to estimate prevalence for an unknown population size. Drug Alcohol Depend Rep..

[b0105] Kilmer B., Everingham S., Caulkins J., Midgette G., Pacula P., Reuter P., Burns R., Han B., Lunderg R. *What America's Users Spend on Illegal Drugs.* 2014.

[b0110] Krishnaswami S., Mukhopadhyay S., Markus S.A., Nechuta S.J. (2022). Prescription Opioid Characteristics and Nonfatal Overdose Among Patients Discharged from Tennessee Emergency Departments. J Emerg Med..

[b0115] Lumley, T. *Survey: Analysis of Complex Survey Samples* [computer program]. Comprehensive R Archive Network (CRAN)2014. https://cran.r-project.org/web/packages/survey/. Last accessed October 26, 2022.

[b0120] National Institute on Drug Abuse. Opioid Overdose Crisis. 2020; https://www.drugabuse.gov/drug-topics/opioids/opioid-overdose-crisis. Last accessed October 26, 2022.

[b0125] Nechuta S.J., Tyndall B.D., Mukhopadhyay S., McPheeters M.L. (2018). Sociodemographic factors, prescription history and opioid overdose deaths: a statewide analysis using linked PDMP and mortality data. Drug Alcohol Depend..

[b0130] O'Donnell J., Gladden R.M., Mattson C.L., Hunter C.T., Davis N.L. (2020). Vital Signs: Characteristics of Drug Overdose Deaths Involving Opioids and Stimulants - 24 States and the District of Columbia, January-June 2019. MMWR Morb Mortal Wkly Rep..

[b0135] Opioid Surveillance Team. Opioid Prevention Program – Data to Action: Overdose Deaths Involving Cocaine With and Without Opioids in New York State Department of Health. Office of Public Health Practice and Office of Drug User Health 2010–2019. New York State ed2021.

[b0140] Preston K.L., Epstein D.H. (2011). Stress in the daily lives of cocaine and heroin users: relationship to mood, craving, relapse triggers, and cocaine use. Psychopharmacology (Berl)..

[b0145] *R: A language and environment for statistical computing. R Foundation for Statistical, Computing* [computer program]. 2020. http://www.R-project.org/. Last accessed October 26. 2022.

[b0150] Reyes-Gibby C.C., Anderson K.O., Todd K.H., Miner J. (2016). Risk for Opioid Misuse Among Emergency Department Cancer Patients. Acad Emerg Med..

[b0155] Roxburgh A., Nielsen S. (2022). Twenty-year trends in pharmaceutical fentanyl and illicit fentanyl deaths, Australia 2001–2021. Int J Drug Policy..

[b0160] Schepis T.S., De Nadai A.S., Ford J.A., McCabe S.E. (2020). Prescription opioid misuse motive latent classes: outcomes from a nationally representative US sample. Epidemiol Psychiatr Sci..

[b0165] Schepis T.S., Wastila L., Ammerman B., McCabe V.V., McCabe S.E. (2020). Prescription Opioid Misuse Motives in US Older Adults. Pain Med..

[b0170] Shiels M.S., Freedman N.D., Thomas D., Berrington de Gonzalez A. (2018). Trends in U.S. Drug Overdose Deaths in Non-Hispanic Black, Hispanic, and Non-Hispanic White Persons, 2000–2015. Ann Intern Med.

[b0175] Substance Abuse and Mental Health Services Administration. 2002-2018 National Survey on Drug Use and Health Public Use File Codebook. In. CBHSQ Methodology Report: Center for Behavioral Health Statistics and Quality, Substance Abuse and Mental Health Services Administration. 2019.

[b0180] Taylor J., Pardo B., Hulme S., Bouey J., Greenfield V., Zhang S., Kilmer B. (2021). Illicit synthetic opioid consumption in Asia and the Pacific: Assessing the risks of a potential outbreak. Drug Alcohol Depend..

[b0185] Zou G. (2004). A modified poisson regression approach to prospective studies with binary data. Am J Epidemiol..

